# Dried Blood Spots in Neonatal Studies: A Computational Analysis for the Role of the Hematocrit Effect

**DOI:** 10.3390/ph16081126

**Published:** 2023-08-10

**Authors:** Chrysa Daousani, Vangelis Karalis, Yannis L. Loukas, Kleopatra H. Schulpis, Konstantinos Alexiou, Yannis Dotsikas

**Affiliations:** 1 Laboratory of Pharmaceutical Analysis, Department of Pharmacy, National and Kapodistrian University of Athens, Panepistimiopolis, 157 84 Athens, Greece; 2 Laboratory of Biopharmaceutics-Pharmacokinetics, Department of Pharmacy, National and Kapodistrian University of Athens, Panepistimiopolis, 157 84 Athens, Greece; 3 Institute of Child Health Agia Sofia Children’s Hospital, 115 27 Athens, Greece; 4 Private Practice, Meg. Alexandrou 16, 145 69 Anixi, Greece

**Keywords:** dried blood spots, hematocrit effect, neonates, screening, pharmacokinetic, computational analysis, Monte Carlo simulations, cut-off

## Abstract

Dried blood spot (DBS) microsampling is extensively employed in newborn screening (NBS) and neonatal studies. However, the impact of variable neonatal hematocrit (Ht) values on the results can be a source of analytical error, and the use of fixed Ht for calibration (Ht_cal_) is not representative of all neonatal subpopulations. A computational approach based on neonatal demographics was developed and implemented in R^®^ language to propose a strategy using correction factors to address the Ht effect in neonatal DBS partial-spot assays. A rational “tolerance level” was proposed for the Ht effect contribution to the total analytical error and a safe Ht range for neonatal samples, where the correction of concentrations can be omitted. Furthermore, an “alert zone” for a false positive or negative result in NBS was proposed, where the Ht effect has to be considered. Results point toward the use of Ht_cal_ values closely representative of populations under analysis and an acceptable level of percentage relative error can be attributed to the Ht effect, diminishing the probability of correction. Overall, the impact of the Ht effect on neonatal studies is important and future work may further investigate this parameter, correlated to other clinical variables potentially affecting results.

## 1. Introduction

Inherited metabolic disorders include a wide range of hereditary conditions that interfere with the body’s metabolism. Most people with such diseases have a gene defect leading to enzyme insufficiency. A large number of different genetic metabolic disorders exist, varying in symptoms, treatment manifestations, and disease courses. To decrease mortality rates and enhance prognosis, recognition of such disorders has to take place as early as possible for timely diagnosis and effective clinical intervention to be achieved. Newborn screening (NBS) is an essential and acknowledged public health tool that takes place in numerous territories worldwide and aims to test all newborns for a growing number of usually inherited metabolic disorders that require suitable therapeutic measures to prevent or mitigate negative health outcomes [1]. The screened conditions differ between regions based on ethnic and economic factors as well as on national health policies [2,3].

Dried blood spot (DBS) constitutes a microsampling technique, employed in several fields of life sciences and drug research [4], in which a very small amount of capillary or venous blood is collected on specific filter paper. This technique is mostly utilized in laboratories performing NBS tests due to its well-established advantages. Indeed, DBS was first introduced more than half a century ago, when Robert Guthrie applied phenylketonuria testing [5], a milestone methodology that marked the initiation of NBS. Throughout time, more disorders were added to NBS programs worldwide, with thyroid-stimulating hormone (TSH) measurements being the second most widely implemented for the diagnosis of congenital hypothyroidism [6]. The evolution of tandem mass spectrometry (MS/MS) technology during the 1990s and onward led to multiplicative analytical concepts where several analytes/metabolites could be screened simultaneously at the same DBS sample [7,8]. Other diseases being widely screened nowadays include congenital adrenal hyperplasia, biotinidase deficiency, galactosemia, aminoacidemias, cystic fibrosis, hemoglobinopathies, glucose-6-phosphate dehydrogenase deficiency, and Duchenne muscular dystrophy. Mutational analyses may also be conducted, providing evidence on the genetic background of the disorders [9,10,11]. Most recently, artificial intelligence and machine learning tools have been also proposed in NBS analyses [12].

In addition to its acknowledged and extended application in NBS, DBS sampling is also gaining more interest in neonatal clinical studies. Among them, applications in therapeutic drug monitoring (TDM) [13] and pharmacokinetic (PK) trials [14,15] are of great importance. The advantages of DBS make it a great technique for all age groups of newborns (including preterm and term neonates) to collect, transport, and store blood for laboratory research. Regarding sampling procedures, a heel puncture is considered the “golden standard”. As far as the maximum allowed blood volume that is permitted to be drawn within a certain time period in neonatal trials is concerned, there are different recommendations reported in the scientific literature [16]. Of note is that the European Medicines Agency (EMA) recommends that the trial-related blood loss should not exceed 3% of the total blood volume during a period of four weeks and should not exceed 1% at any single time, per individual [17]. Considering that total blood volume is estimated at 80 to 90 mL/kilogram body weight, DBS is the ideal sampling methodology for the neonatal population. The dynamic evolution and widened acceptance of DBS in the field of bioanalysis have also been recently acknowledged in the recent International Council for Harmonization of Technical Requirements for Pharmaceuticals for Human Use (ICH) Bioanalytical Method Validation Guidelines [18], where dried matrix techniques—when adequately validated—are considered appropriate to support regulatory applications and are discussed in such a framework for the first time.

Despite the many benefits connected with this alternative sampling approach, there are still several difficulties, with the so-called ‘hematocrit (Ht) effect’ being, beyond a doubt, the most discussed issue [19,20]. Hematocrit is defined as the ratio of the volume of red blood cells to the total volume of blood. Several factors such as age, ethnicity, gender, dietary, and pathological conditions affect the measured Ht values [20,21]. It is well-known that Ht impacts the blood viscosity, thus affecting the spreadability of blood onto DBS filter paper, with samples presenting high Ht being more viscous compared to those with low Ht. The prospective impact of Ht on DBS assay parameters (accuracy and precision, matrix effects, and analyte recovery) is well-defined and reviewed in the literature as well as the numerous strategies and technologies developed to address the Ht effect [19,20,22]; the most recent approaches include the utilization of capillary electrophoresis [23], spectroscopic methods for hemoglobin normalization [24], patterned DBS cards [25], and Ht prediction of DBS using near-infrared (NIR) spectroscopy [26]. Obviously, if the Ht values of the unknown blood samples differ considerably from the Ht value of the calibration standards, assay bias is certainly affected; to assure the quality of DBS bioanalytical methods, evaluation of the Ht impact should be part of the validation process [18,21].

One of the strategies applied to address the Ht effect is to correct quantified concentrations with a suitable correction factor (i.e., equations integrating bias corrections through the correlation of Ht to a physical parameter). A computational analysis concentrated on the use of DBS for quantitative bioanalysis in adult research was proposed in our previous work [27], providing a well-defined structure for the correction of the Ht effect by utilizing correction factors. Specific Ht calibration values were suggested, rationalized on the basis of demographic data (i.e., the use of a Ht calibration value representing the study population). A maximum level for percentage relative error was proposed as a rational contribution of Ht to the percentage total analytical error, reducing the need for concentration correction, so that the general regulatory criteria for bioanalysis may be met.

However, the background and parameters affecting neonatal DBS measurements present substantial differences compared to the adult population. First of all, in comparison to older children and adults, neonates have significantly higher Ht levels, since values of 50–55% are usually observed in newborns during the first days of life [28]. NBS calibrators are therefore created with such Ht, since screening is usually conducted within the first 24–72 h of life [29,30]. Furthermore, in neonates, time dependency and significant variability of Ht values are noted [28]. Gestational age and birth weight, various health conditions (including anemia and polycythemia) as well as feeding practices (time and type of feeding, e.g., parenteral, per-os, or full breast-feeding), are important factors that may affect neonatal Ht values.

Furthermore, DBS sampling and analysis for NBS are based on different principles compared to neonatal assays (e.g., TDM or PK/clinical measurements). The screening of various metabolites and disease biomarkers in NBS programs employing MS/MS is realized through pre-defined “cut-off” values that are population-based and clinically validated [10,31]. A significant challenge in NBS is therefore the case of borderline results; that being said, a newborn sample measured close to the cut-off value (slightly lower or higher) leads to an indistinguishable difference between a positive and a negative test outcome. In such cases, analysis has to be repeated before definite conclusions can be drawn regarding any further steps, and additional diagnostic tests may be applied to assist in the clinicians’ diagnosis. Obviously, in a screening test, such false-positive or false-negative results may not be avoided, leading to significant stress for the families [32].

Currently, Ht is not taken into account for the establishment of NBS cut-off values. The elimination of the Ht effect seems like a definite need in order to avoid bias. In fact, Ht effects on the analysis of NBS diagnostic markers have been reported in the literature [33,34,35,36,37]. Furthermore, it appears that the effect of Ht on the analysis of NBS diagnostic markers is analyte-specific. Therefore, for NBS, a Ht-related bias elimination strategy may include setting screening “cut-off” values that take into account the physiological Ht variability or, in other words, defining an “alert zone” for a false-positive or false-negative result, where the Ht effect has to be considered.

On the other hand, DBS assays used in clinical or PK studies in neonates may also be affected by the time-dependency and Ht variability observed the first days after birth for both term and preterm neonates. Most importantly, due to the difficulties and ethical obstacles for using classical sampling (i.e., multiple blood draws) in such studies, DSB samples are usually coupled with population PK modeling techniques; this allows for the estimation of the PK parameter values through sparse data [38]. However, population PK analysis identifies parameters that have a significant impact on drug concentrations and may account for part of the variability. Consequently, the numerous sources of variability increase the possibility of surpassing the currently applicable acceptance criteria for the total amount of bias for validated assays in regulated bioanalysis for these studies. Of note, a ±15% (percentage bias) and ±20% for the lower limit of quantitation (LLOQ) are the accuracy criteria accepted by ICH for chromatographic assays [18]. The literature data show that the Ht effect may be a concern for PK parameter estimation in neonatal studies, and thus it has to be definitely considered for DBS method validation and following the population PK modeling exercise [39,40,41].

This work focused on defining a rationalized strategy to address the Ht effect in DBS neonatal studies. The aim was to try and set acceptable bias limits where concentration corrections may be omitted, relying on computational methodology and taking into account the Ht demographic peculiarities of the neonatal population as well as the different perspectives of NBS in contrast to PK or clinical studies in this vulnerable population.

## 2. Results

**Figure 1:**
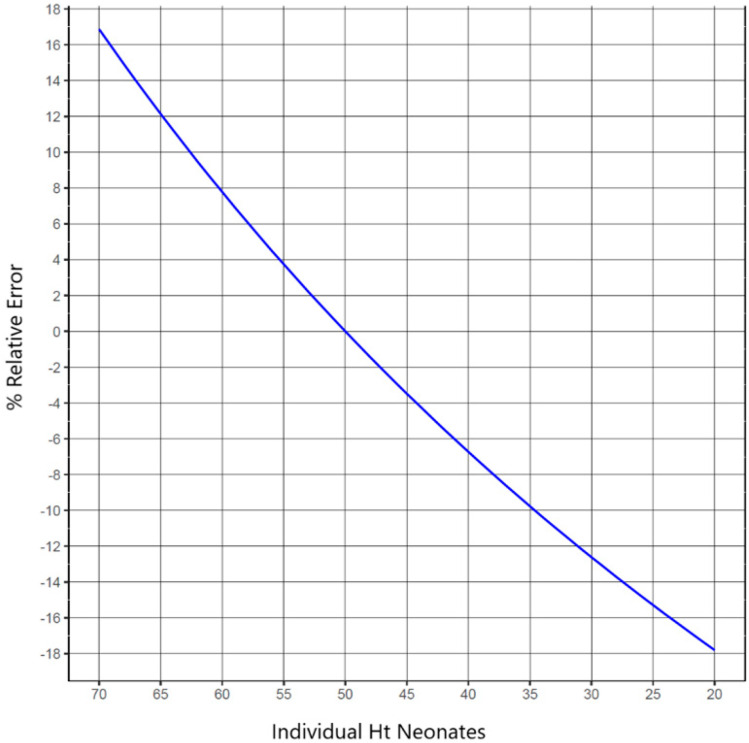
Percentage relative error as a function of the individual neonates’ Ht values.

In order to investigate the Ht influence on DBS assays (i.e., the Ht value applied for calibration purposes and its effect on the percentage relative error in the analytical setting of samples with different Ht), the already portrayed simulation-based analysis was applied. Figure 1 depicts the percentage relative error vs. individual neonate Ht values for a given Ht_cal_ value (Ht_cal_ was set equal to 50%) by utilizing Formula (1) [27] through the simple modeling approach already described. Formula (1) is a linear equation: y = −0.0070x + 1.32 (R^2^ = 0.984), generated for the correlation of a blood dispersion measure with Ht values. y is the DBS area (cm^2^) and x is the sample’s Ht value. The utilized paper was Whatman 903™ and the blood volume applied was 50 μL. Based on the above equation, knowledge of the Ht value of the unknown sample can permit calculation of its area. Following this, a sample-specific correction factor can be estimated by using the areas’ ratio (unknown to standard sample) [27]. For this purpose, the individual Ht range for newborns has been established at 20–70%; as mentioned, this range is thought to include different neonate subpopulations (e.g., preterm or term neonates with Ht reference values presenting variability, neonates with clinical disorders causing low Ht, or even polycythemic neonates with Ht >65%). This is important, since the potential Ht differences between neonates are not only attributed to the comparison between healthy babies and patients in certain disease states, but also between neonates of different gestational ages (preterm and term) as well as neonates of different post-natal age (as discussed, Ht varies greatly in the first days of life). This variability may pose a negative impact on the interpretation of data across studies or the utilization of DBS methods to PK and clinical studies and of course, on NBS. Indeed, the Ht effect on the physical characteristics of DBS samples, and subsequently on the accurate quantification of analytes within these samples, is significant if the Ht levels of study blood samples are anticipated to vary widely (especially outside the range considered to be “normal” for each subpopulation) or to differ significantly from those of the calibration standards. Due to the Ht effect, results from such studies may be based on different measured concentrations for the same analytes. According to the results of this analysis (blue line in Figure 1), the percentage relative error attributed only to Ht, runs from −17.80 to 16.87 within the selected Ht range of 20–70% and for the specified Ht_cal_ value of 50%. In other words, to obtain a relative error of ±5%, the Ht of individual samples will have to fall within the range of 42.71–56.60% for a Ht_cal_ set at 50%. It should be noted that these numbers may fluctuate for real samples, and real observations may differ from those theoretically predicted due to the fact that a variety of factors affect the total error.

**Figure 2:**
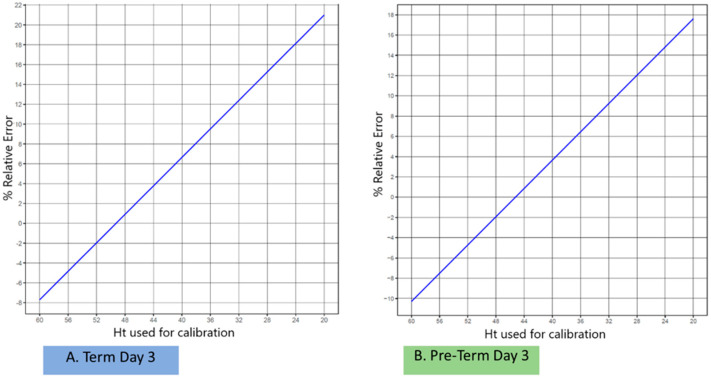
Percentage relative error versus the Ht value used for calibration (Ht_cal_). Panel (**A**): Simulations for term neonates at post-natal day 3; Panel (**B**): Simulations for preterm neonates at post-natal day 3. Under each point, a number of 1,000,000 Monte Carlo studies were performed.

As a subsequent goal, Monte Carlo simulations were applied, aiming at recommending specific Ht_cal_ values on the basis of the demographics of the newborn babies (Figure 2). By establishing a “tolerance level” of the Ht effect to the percentage total analytical error, it is then possible to define a plausible Ht range for the measurement of the concentrations of the neonate samples, where the correction of concentrations of unknown samples may be avoided. However, as already mentioned, in contrast to adults, neonates present a variable Ht depending on both the gestational and post-natal age. Therefore, simulations were performed by taking into account different neonate sub-groups (of different gestational ages) as well as selected timepoints (days after birth) for sampling; the set parameters are presented in Table 1. Figure 2 depicts the percentage relative error correlated to Ht_cal_, where simulations were performed for term and preterm neonates at post-natal day 3; the same exercise was performed for all combinations of the gestational age and post-natal age selected milestones shown in Table 1. For all neonatal populations including males and females equally, the Ht_cal_ levels were set at a range of 20–60%; the assumed Ht_cal_ values were taken into consideration based on the reference values acquired from Jopling et al. [28] and, once more, Formula (1) [27] was applied. When Ht_cal_ was set at the mean value for each distinct population, it led to differentiation of the two groups on the same post-natal day, as can be seen from a visual observation of Figure 2; this means that the 0% relative error was achieved when Ht_cal_ was ideally set at a different value for each subpopulation and not by considering a fixed Ht_cal_ (e.g., 50% for all neonates). Similar observations were obtained for all neonate sub-groups investigated, revealing that the ideal Ht_cal_ for neonatal studies has to be considered on a case-by-case basis, taking into account both gestational age as well as the post-natal day of sampling for a given population to be analyzed.

Table 1 was created using the whole set of simulations in order to determine the Ht level up to which a specific percentage of relative error occurs; mathematical Formula (1) [27] was again used for the estimations. The applied Ht_cal_ values were based on the literature-obtained reference values and assumed as the mean for each specific neonate subpopulation. As gender was not observed to influence Ht in neonates [28], equal representation of males and females was assumed for all simulations. In order to explicate the investigation that was conducted, considering the outcome of the modeling exercise (Figure 1), the set percentage relative error values, namely 1%, 3%, and 5%, were arbitrarily determined. Obviously, only part of the total analytical error can be due to the Ht effect; a reasonable 5% upper “acceptance” level of the Ht effect may be assumed, for which the Ht-allocated error may be ‘tolerated’; a lower percentage relative error may be less realistic for neonates, taking into account the higher variability of Ht for such populations.

The respective Ht range was then calculated for each subpopulation using Equation (1) [27], aiming to not overpass the set percentage relative error values. The impact of Ht is anticipated to be insignificant or minimal in this range of values, which reflects a ‘tolerance’ percentage error limit where correction may be avoided. Based on the analysis performed, a reasonable ±5% relative error threshold may be suggested; obviously, any sound and well-justified approach to this proposed limit may be driven by different factors and experimental parameters. For example, in contrast to the utilization of DBS sampling for PK studies or TDM purposes, where a calibration curve is constructed, NBS is, in most laboratories, based on a single-point calibration, and is therefore less prone to analytical error; that being said, a less strict approach may be applied in such cases.

Overall, this work may serve as the basis for the consideration of utilizing the well-acknowledged demographics for the different neonate subpopulations (based on both gestational as well as post-natal age) and setting a Ht_cal_ value for the preparation of calibration samples at a Ht reflecting the time-studied population. In this way, neonatal DBS accuracy will not be largely affected by the Ht effect, which cannot be avoided in cases where a fixed Ht_cal_ is set, and is not representative for all neonates.

**Figure 3:**
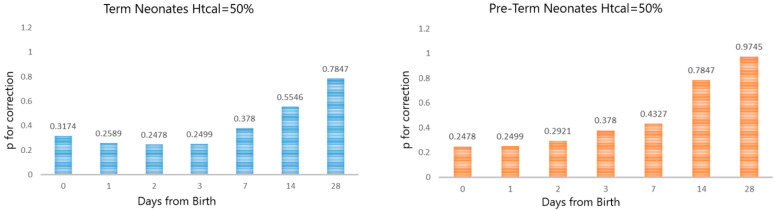
Graphical presentation of the probability of correction (*p*) in relation to the different neonate subpopulations and sampling times, assuming a fixed Ht_cal_ of 50% and an indicated “tolerable” percentage relative error of ±5%.

Furthermore, the so-called probability (*p*) for correction, related to the different neonate subpopulations and sampling times, assuming a fixed Ht_cal_ value set at 50% and a “target” analytical error of ±5%, is depicted in Figure 3. Although it is well-acknowledged that the skewing of Ht distributions may be a fact, the consideration of the normal distribution of the individual Ht values for the various neonate populations of gestational and post-natal ages was set as an assumption in order to simplify the investigation. By observing Figure 3, it can be seen that for the various post-natal sampling timepoints of both the term and preterm neonates, a set Ht_cal_ 50% led to the need for correction for a different proportion of cases.

Overall, a reasoned choice of Ht_cal_ value leads to a lower chance of needing correction; conversely, a Ht_cal_ value that does not ‘reflect’ the studied population leads to an increased risk of concentration correction necessity. Of course, neonates are considered as a population with a large variability in Ht values, meaning that a full avoidance of the need for correction seems unlikely. However, by carefully selecting the Ht_cal_ based on subpopulation characteristics (and knowledge of the Ht values of the samples), we may safely reduce this probability.

The last part of the current work was related to the investigation of the Ht effect on the DBS measurements in NBS applied to a series of 10,018 neonatal samples. By observing the obtained results for each analyte, the number of definite positive cases (i.e., the samples exceeding the pre-defined “cut-off” limit) as well as the number of samples with experimental concentrations being measured within the “cut-off” value and a value set at the 10% difference from the defined “cut-off” were identified (Table 2).

**Figure 4:**
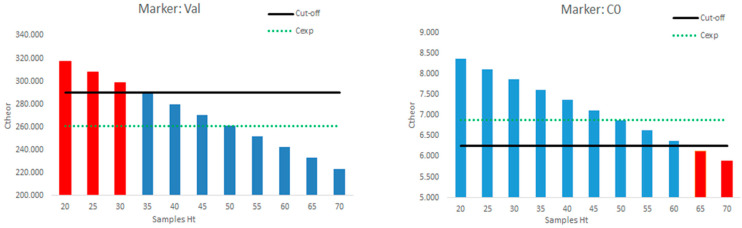
Graphical presentation of the Ht effect on the NBS results for specific markers (Val: valine; C0: free carnitine; C_exp_: experimental (initial) concentration; C_theor_: theoretical (corrected) concentration). C_exp_ was assumed to be 10% different of the respective cut-off limit. Ht_cal_ was set at 50%.

The realistic influence of the Ht effect on the NBS experimental values and the need for correction are presented in Figure 4 for two selected disease markers; the same analysis was undertaken for all markers in Table 2 with similar results and observations. Figure 4 depicts a graphical correlation of the individual samples’ Ht versus the theoretical (corrected concentration) when the samples’ respective experimental value was set at the 10% difference from the established “cut-off”; this means that such samples are considered to be negative based on the obtained analytical measurements. Again, the exercise was undertaken considering a fixed Ht_cal_ of 50%.

However, by observing the data for the marker valine, it is evident that for a sample with low Ht (<35%), as could be the case for anemic neonates, the corrected concentration would exceed (being higher) the “cut-off” limit (red columns). In contrast, for the marker C0, where positives are considered when being lower than the “cut-off” value, samples with high Ht (over 65%, e.g., in polycythemic neonates) would result in corrected concentrations, leading to the samples’ characterization as positive. Overall, based on the demographics of different neonate subpopulations (Table 1) it is evident that, at least for otherwise healthy newborns where the respective Ht reference values are applied, the usual NBS sampling timepoints (within the first 3 days) will not lead to false-positive results for experimental concentrations at the 10% difference set for this exercise, even by applying a set Ht_cal_ of 50%. However, for neonates with extreme Ht values, the Ht effect will largely influence the outcome and the concentration correction will lead to alteration of the sample’s characterization from negative to positive.

## 3. Discussion

The objective of this study was to examine the impact of Ht on DBS partial-spot assays, which are designed for use in neonatal screening, PK analysis, and clinical investigations. Additionally, a potential approach for correcting this effect was proposed. The latter approach was founded on the utilization of correction factors, as previously suggested in our prior research endeavors.

DBS sampling is an alternative collection technique where a blood sample is applied onto a specific paper card. This methodology is currently applied worldwide for sample analyses in neonatal studies, with NBS being the most prominent. One significant parameter influencing DBS is the Ht value in blood, which affects the blood distribution on the filter paper card and, in turn, impacts the validity of the DBS results (e.g., drying time, homogeneity of spot, accuracy, and precision of assays). For this reason, correction is needed for the Ht concentration, and different strategies have been developed to minimize the influence of Ht. This is of particular concern in neonates, who have higher Ht levels compared to other age groups and show a large variability in Ht, as this is affected by both the gestational and post-natal age [28,42].

Gestation progress positively affects Ht while, after birth, a prominent reduction in Ht within the first 28 days of life takes place; this neonatal anemia is a physiological phenomenon that is attributed to the different oxygen levels between the intrauterine and extrauterine environments. This reduction reaches an end at about the 6th to 12th week of life, being followed by an increase, which leads to Ht values similar to those of adults up to the age of 2 years [43,44,45]. The situation is somehow different for preterm infants, where lower Ht values are, in general, documented at birth and a more rapid reduction is observed within the first weeks of life [43,44,45,46,47,48]. In any case, especially for these small neonates, iatrogenic processes applied during their hospitalization (such as blood transfusions or blood loss due to frequent laboratory testing) affect their Ht values. On the contrary, up to 5% of the total neonatal population is affected by a condition called polycythemia, which refers to unusually high Ht values (e.g., more than 65%) [49,50,51]. This condition is affected by factors such as gestational age and birth weight; it is also more frequent in high altitudes [50].

Taking into account the above, it is evident that Ht presents high variability in neonates, and in some specific subpopulations, the Ht values may be extreme. Ht reference ranges have been published; for term neonates (including the so-called late preterm infants, meaning overall 35–42 weeks of gestation), this range at birth (5th to 95th percentile) is 41% to 65% (mean: 53%) as well as for premature babies (29–34 weeks of gestation), whose respective values are somehow lower (38% to 62%, mean: 50%) [28]. As a usual practice, since a hematocrit of approximately 50% may be generally anticipated within the first post-natal days, calibration materials for NBS or other neonatal studies are prepared with such Ht values [29]. However, such a fixed Ht_cal_ value may not be representative of the values obtained at different sampling timepoints (e.g., samples taken at different post-natal ages) or the different neonatal subpopulations (term or preterm), or even of the babies’ clinical condition; the latter is very important since DBS analysis may apply in these groups not only for NBS, but also as part of PK or clinical trials or for TDM purposes. Actually, as revealed in the present work, the average Ht, even for a healthy term baby within the first month of life, let alone a preterm or critically ill neonate, may ordinarily be less than 50% (Table 3), and a lower H_tcal_, depending on the post-natal day of sampling, could be more appropriate to set in order to avoid a potential effect on the results. On the other hand, for polycythemic neonates with significantly higher Ht values at the time of screening, a higher Ht_cal_ may be necessary.

NBS programs worldwide are based on population-defined “cut-off” values for different metabolites in DBS samples, which are typically obtained on the third day of life. A sub-punch of specific size is retrieved from the DBS and extraction of the biomarkers takes place before analysis. In cases where the disease marker experimental concentrations largely exceed their respective “cut-off” levels, Ht may not affect decision making, irrespective of the need for concentration correction. However, NBS-led diagnosis may be complicated in cases where metabolite concentrations are only marginally outside their corresponding “cut-off levels”. Obviously in such cases, the Ht effect on the metabolite concentrations may positively or negatively influence decision making, and thus the diagnosis. Our analysis, which is based on the demographics of different neonate subpopulations combined with both their gestational as well as post-natal age, showed that, at least for otherwise healthy newborns where the respective Ht reference values were applied, the usual NBS sampling timepoints (within the first 3 days) will not lead to false-positive results for experimental concentrations at a 10% difference set for this exercise, even by applying a fixed Ht_cal_ of 50% followed by subsequent correction of the concentrations. However, for neonates with extreme Ht values, the Ht effect will largely influence the outcome and the application of the correction factor will lead to an alteration in the sample’s characterization from negative to positive.

The DBS approach has more recently gained interest in other neonatal studies such as PK trials or TDM. Despite the fact that the need to obtain PK data in children is crucial, the conduction of such studies presents ethical constraints and methodological challenges, mostly related to frequent sampling requirements. In this context, respective guidelines worldwide recommend that trial-related blood loss should not exceed a specific percentage of the total blood volume within pre-specified time periods, and neonatal studies should be designed and performed taking these limitations into account. DBS samples coupled with population PK modeling techniques have been shown to be an appropriate methodology in this respect, since they allow the use of low sampling volumes and sparse data [14,39]. Ht has been defined as a factor potentially affecting the DBS concentration measurements, and thus the obtained PK parameter values [38,40].

Therefore, there is a reasonable rationale for developing strategies to address the Ht effect in neonatal DBS assays with partial-spot analysis, both within the margins of NBS as well as in other studies. Indeed, the results of the present research, which are in agreement with our previous work, prove that, also for neonatal studies, the use of a Ht_cal_ value based on demographic data is a rational approach that will result in an “acceptable” level of percentage relative error attributed to the Ht effect. However, the neonate population is not homogeneous, and due to the given variability in the Ht values, an upper level of 5% for relative error could be a ‘tolerable contribution’ of the Ht effect to the percentage total analytical error in most cases, so that well-acknowledged control of the Ht effect can be achieved, and compliance to the recommendations of the bioanalytical guidelines can be met. Other important factors affecting DBS performance such as the involvement of experienced laboratory personnel, the utilization of calibrated equipment, optimization of the extraction process, homogeneity of spots for the analyte(s) tested in partial DBS analysis protocols, the Ht influence on analyte recovery and matrix effects also have to be considered during method validation. Addressing factors that affect DBS validation such as the Ht effect will facilitate the NBS process, but will also enhance the regulatory acceptance of the DBS platform for PK and clinical studies, as also foreseen in the recent ICH Bioanalytical Guidelines [18].

The present study sets forth a logical “tolerance level” for the Ht effect’s contribution toward the overall analytical error. Furthermore, it suggests a secure range of Ht values for neonatal samples, within which the need for concentration correction can be obviated. Additionally, this study has endeavored to establish a designated “alert zone” in newborn screening for instances of false-positive or false-negative outcomes, necessitating the consideration of the Ht effect. The aforementioned outcomes were attained through the implementation of a computational approach and the utilization of demographic data pertaining to neonates, thereby highlighting the notable fluctuations in Ht measurements. The Ht of neonates is influenced by both their gestational and post-natal age, rendering the application of a uniform Ht_cal_ value unsuitable for all subgroups of neonates. The significance of this matter is particularly noteworthy for NBS due to the potential occurrence of erroneous outcomes, either false-positive or false-negative, in neonates with significantly elevated or reduced Ht levels. Our findings suggest that utilizing Ht_cal_ values accurately to reflect the populations being studied can lead to a tolerable degree of relative error associated with the Ht effect. This approach can reduce the likelihood of requiring correction. In general, the significance of the Ht effect on neonatal research is noteworthy, and forthcoming endeavors could be directed toward exploring this parameter in conjunction with other clinical factors that may influence outcomes via substance–covariate associations.

It is acknowledged that there are some limitations within the present work, starting with the restrictions posed due to the available Ht reference ranges; firstly, the inclusion of preterm infants <29 weeks of gestational age could not be conducted within this analysis, since the respective Ht reference ranges for this subgroup are not available [28]. Non-availability of respective reference values also stands for critically ill neonates and for those who were given a blood transfusion. A second limitation was that the published Ht reference values utilized for this work represent tested blood samples from capillary, venous, or arterial origin [28]; this may have had some impact on the Ht values obtained. Furthermore, the reference ranges that were applied in this study are considered to be representative to those expected at sea level; application of the findings in studies at high altitudes may not be representative due to the impact of altitude on Ht being recognized in scientific knowledge [28].

Another issue for discussion is the fact that blood in DBS sampling differs substantially from the serum or plasma samples usually analyzed in PK or clinical studies. In order to correlate whole blood to plasma concentrations when DBS is applied in such studies, in addition to classical liquid sampling, cross-validation should take place [52]. As differences in Ht are considered as extremely important factors that affect the so-called ‘blood-to-plasma ratio’ of a drug [27], DBS-based quantitative analysis or TDM of many categories of medicines is shown to be influenced by Ht [20,53], depending on their different distribution in plasma and red blood cells. For this reason, in DBS-based neonatal studies for TDM purposes, the Ht-effect was also considered [41].

The current analysis discussed the Ht-related error attributed to differential blood viscosity/dispersion. However, other confounding factors may affect the NBS biomarker analysis, the difference of concentration between plasma and cellular compartments being an important one, as already mentioned. Indeed, reports in the scientific literature show that, since distributions in plasma and red blood cells differ for each metabolite, Ht affects the NBS analysis of several amino acids and acylcarnitines [29,34,54,55]. For this reason, Ht is also reported to have significant effects on the DBS analysis of TSH [37], as the hormone is contained only in plasma, meaning that the higher the sample Ht, the lower the TSH concentration. These findings do not suggest that the existing NBS methodologies should be re-considered, as they rely on “cut-off” values obtained through thorough and long-term population research. On the other hand, when evaluating the results of neonates with extreme Ht values, these observations may be thought of as an extra significant reason to be mindful of the Ht influence on NBS analysis. When the newborn screening samples are collected, newborns who have severe polycythemia or anemia may be re-examined using a Ht_cal_ value based on their demographic profile. Another co-factor of potential impact results from the observation that the NBS sampling time was recently seen to affect concentrations of disease markers (sampling points investigated were between 12 to 72 h following birth). Such observations led to the conclusion that the metabolic processes in early neonatal age may be subject to time dependence, and subsequently, a false-positive diagnosis may be set for some metabolic conditions [30]. As the date of sampling is also related to Ht, this co-factor may act additively or conversely to the Ht-related error.

Overall, a deeper understanding can be obtained through future research of the method- or substance-related factors and their potential additive or opposite influence on the Ht-effect in neonatal DBS analysis. For example, the correlation of the Ht-related error to the Ht influence on the analytes’ concentrations due to their variable blood-to-plasma distribution, the latter being analyte-specific, or to the potential impact of the NBS sampling time on disease marker concentrations could be the subject of interesting future investigations aiming to define the overall contribution to the total error of these factors.

## 4. Materials and Methods

As the first step of this work, the principal target was to suggest specific Ht values for the preparation of standards when the analysis of the neonate samples takes place, where the correction of concentrations of unknown samples may be omitted. The applied methodology relies on published demographic data and is based on the modeling approach that was previously developed for adult sampling [27]. The computation work is based on the principle of correction through the use of specific, linear-type mathematical formulas correlating a dependent variable of blood spreading with the measured Ht. The current work was performed with the utilization of the mathematical Formula (1), as derived and described in our laboratory’s previous research [27,56]. The computational approach included a simple modeling component as well as Monte Carlo simulations with the creation of virtual neonate populations with different Ht levels that was implemented. All statistical analyses were performed in the R^®^ language (version 4.3.1) [57].

It should be mentioned that for neonates, the demographic data on Ht values present specific differentiations compared to adults [58]. First of all, due to the obvious difficulties and ethical constraints, newborn Ht normal values (i.e., data obtained from healthy neonates) are not available. Instead, so-called “reference ranges” are published, obtained from neonatal patient populations when blood sampling is performed for specific clinical testing. In our analysis, we utilized the Ht reference ranges for the first 28 post-natal days presented in the literature [28] (mean, 5th, and 95th percentile values), derived from 41,910 neonates; this population was divided into two major groups based on the gestational age at time of birth: term (including “late preterm”) neonates (35–42 weeks of gestation) and preterm neonates (29–34 weeks of gestation). By carefully observing these reference values, it is evident that these are the two major influential factors that affect neonatal Ht, namely, the gestational age as well as the post-natal age, while gender was not found to be an impacting parameter. According to this research [28], the studied neonatal population did not include cases where the mothers presented with specific medical diagnoses such as placental abruption or placenta previa, or the babies diagnosed with a chromosomal disorder, neonatal anemia, or having received a blood transfusion. A limitation of the dataset is that it was not possible to include and analyze preterm infants <29 gestational weeks, since such patients are all subjected to frequent blood samplings and blood transfusions.

Table 3 presents the mean and standard deviation (SD) Ht values extracted from [28] for selected timepoints (days after birth). Data were extracted from Figure 3 in [28], panels A and C, using the WebPlotDigitizer software (version 4.6) [59].

A simple modeling approach was initially applied, which followed the same steps as in our previous work [27]. However, in this study, an assumed Ht_cal_ (i.e., the Ht value used in the calibrators) equal to 50% was used for the simulations, being the usual Ht value that is chosen to prepare calibrators in neonatal studies as a representative value for the first days of life. Again, a series of Ht values for the unknown sample were used in a range of 20–70%, considering the inclusion of extreme cases (e.g., anemic (low Ht) and polycythemic (high Ht >65%) neonates).

Following the above, Monte Carlo simulations were then used to generate virtual neonate populations [27]. The normal distribution was assumed to be followed for Ht values, for which the demographics (means and SD) were based on the reference values from [28]. Simulations were undertaken for a total of 1,000,000 virtual subjects for each of the selected timepoints (post-natal age) and separately for the term and preterm neonate groups, assuming both males and females in equal parts. The percentage relative error was calculated each time as per the methodology in [27], assuming several values for Ht_cal_ (at a range of 20–60%). Furthermore, based on these findings, the individual Ht range for certain percentage relative error values was defined, using Ht_cal_ set at 50% (fixed Ht for calibration) or set at the mean Ht values for term and preterm neonates at specific post-natal days. The probability of correction in relation to the neonates’ different subpopulations, assuming a fixed Ht_cal_ and for an indicated “tolerable” percentage relative error, was finally estimated. The free online statistics tool OnlineStatBook Version 2.0 [60] normal distribution calculator was used for the calculations.

As the second step of the work, the Ht effect in NBS was investigated in order for a Ht-related bias elimination strategy to be proposed. As mentioned, NBS assessment is based on NBS “cut-off” values for respective disease markers, which are laboratory- specific. We applied the “cut-off” values estimated by [10] in order to evaluate a series of 10,018 neonatal specimens obtained via the expanded NBS program in Greece. Subjects were screened for specific metabolic conditions including amino acid metabolic defects, fatty-acid oxidation, and organic acid disorders. The dataset included 33 metabolic analytes/disease markers measured by MS/MS, based on the protocol previously described [61]. By observing the obtained results, a number of definite positive cases could be identified for each analyte; such samples are definitely subject to re-analysis. Subsequently, the number of samples lying between the “cut-off” value and an experimental value calculated at the 10% difference from the set “cut-off” were identified. This interval is arbitrarily set as a reasonable “alert zone” of the experimental concentrations where the Ht effect is then investigated in terms of its potential impact (besides the relative error) on corrected concentrations, and subsequently the need for re-analysis, should these be declared positive.

## 5. Conclusions

DBS analysis has been shown to be a largely advantageous sampling methodology in neonatal analysis compared with traditional liquid samples, mainly due to the need for significantly smaller sampling volumes; the application of DBS to neonatal screening as well as to PK or clinical studies in this vulnerable population is a given fact. However, the impact of variable neonatal Ht values on the results can be a source of analytical error.

The aim of this work was to investigate the Ht-related effect in DBS partial-spot assays intended to be used in NBS, but also in neonatal PK or clinical studies and to suggest a potential correction strategy. The latter is based on the application of correction factors, as already recommended in our past research work. Now, the current analysis proposes a rational “tolerance level” for the contribution of the Ht-effect to the total analytical error, and subsequently, a safe Ht range for neonatal samples, where the correction of concentrations can be avoided. Furthermore, this research also attempted to define an “alert zone” for a false-positive or false-negative result in NBS, where the Ht effect has to be considered. All of the above was achieved with the application of a computational methodology and the use of neonatal demographic data, showing the significant variability of Ht values. For neonates, both the gestational as well as the post-natal age affect the Ht, and therefore, the use of a fixed Ht_cal_ value is not representative of all neonatal subpopulations. This is especially important for NBS, since it may lead to false-positive or false-negative results in neonates with more extreme Ht values. In this vein, our results point toward the use of Ht_cal_ values closely representative of the populations under analysis, and in this way, an acceptable level of percentage relative error can be attributed to the Ht effect, diminishing the probability of correction. Overall, the impact of the Ht effect on neonatal studies is important, and future work may be targeted to further investigate this parameter in correlation to other clinical variables that may affect the results through substance–covariate relationships.

## Figures and Tables

**Table 1:** The Ht range for certain percentage relative error values: ±1%, 3%, and 5% in the case of an entire neonate population and Ht_cal_ set at 50% (fixed Ht for calibration), or with Ht_cal_ set at the mean Ht values for term and preterm neonates at specific post-natal sampling days.

	Level of % Relative Error	Individual Hematocrit	
		Low	High
**Entire Neonate Population (Ht_cal_ = 50)**	1%	48.60	51.37
	3%	45.71	54.04
	5%	42.71	56.60
**Term Neonate Population-Day 0 (Ht_cal_ = 53)**	1%	51.63	54.34
	3%	48.81	56.95
	5%	45.86	59.46
**Preterm Neonate Population-Day 0 (Ht_cal_ = 50)**	1%	48.60	51.37
	3%	45.71	54.04
	5%	42.71	56.60
**Term Neonate Population-Day 1 (Ht_cal_ = 51)**	1%	49.61	52.36
	3%	46.75	55.01
	5%	43.76	57.55
**Preterm Neonate Population-Day 1 (Ht_cal_ = 49)**	1%	47.59	50.38
	3%	44.68	53.07
	5%	41.65	55.65
**Term Neonate Population-Day 2 (Ht_cal_ = 50)**	1%	48.60	51.37
	3%	45.71	54.04
	5%	42.71	56.60
**Preterm Neonate Population-Day 2 (Ht_cal_ = 47)**	1%	45.57	48.40
	3%	42.62	51.12
	5%	39.55	53.74
**Term Neonate Population-Day 3 (Ht_cal_ = 49)**	1%	47.59	50.38
	3%	44.68	53.07
	5%	41.65	55.65
**Preterm Neonate Population-Day 3 (Ht_cal_ = 45)**	1%	43.55	46.42
	3%	40.56	49.18
	5%	37.44	51.84
**Term Neonate Population-Day 7 (Ht_cal_ = 45)**	1%	43.55	46.42
	3%	40.56	49.18
	5%	37.44	51.84
**Preterm Neonate Population-Day 7 (Ht_cal_ = 44)**	1%	42.54	45.43
	3%	39.53	48.21
	5%	36.39	50.88
**Term Neonate Population-Day 14 (Ht_cal_ = 42)**	1%	40.52	43.45
	3%	37.47	46.27
	5%	34.29	48.98
**Preterm Neonate Population-Day 14 (Ht_cal_ = 38)**	1%	36.48	39.49
	3%	33.34	42.39
	5%	30.08	45.17
**Term Neonate Population-Day 28 (Ht_cal_ = 38)**	1%	36.48	39.49
	3%	33.34	42.39
	5%	30.08	45.17
**Preterm Neonate Population-Day 28 (Ht_cal_ = 31)**	1%	29.41	32.56
	3%	26.13	35.59
	5%	22.71	38.50

**Table 2:** Mean metabolite values following the screening of 10,018 neonates; the number of positive cases and the number of cases with values measured between the cut-off and a 10% difference are presented.

Analyte (Marker) ^1^	Cut-Off Value (μmol/L) [10]	Number of Positive Cases	10% Difference from Cut-Off Value (μmol/L)	Number of Cases with Values from Cut-Off up to 10% Difference
**↑Val**	290.000	5	261.000	9
**↑Leu**	245.000	39	220.500	80
**↑Met**	63.000	63	56.700	54
**↑Phe**	153.000	1	137.700	1
**↑Tyr**	190.000	92	171.000	57
**↑Glu**	560.000	95	504.000	48
**↑Orn**	250.000	43	225.000	26
**↑Cit**	65.000	6	58.500	2
**↑Arg**	100.000	13	90.000	8
**↑Ala**	900.000	1	810.000	1
**↑Gly**	1000.000	6	900.000	12
**↓C0**	6.250	38	6.875	38
**↑C3**	7.000	29	6.300	31
**↑C4**	1.690	1	1.521	1
**↑C4-OH**	0.550	27	0.495	40
**↑C5**	1.000	1	0.900	1
**↑C5:1**	0.450	3	0.405	3
**↑C5-OH**	1.040	6	0.936	1
**↑C6DC**	0.270	145	0.243	95
**↑C6**	0.470	9	0.423	10
**↑C8**	0.350	126	0.315	103
**↑C10:2**	0.500	5	0.450	5
**↑C10:1**	0.300	187	0.270	133
**↑C10**	0.420	41	0.378	36
**↑C3DC**	0.250	284	0.225	142
**↑C5DC**	0.300	156	0.270	155
**↑C14:1**	0.640	6	0.576	3
**↑C14**	0.810	12	0.729	20
**↑C16:1**	0.340	289	0.306	311
**↑C16**	9.280	1	8.352	4
**↑C18:1**	3.000	8	2.700	9
**↑C16-OH**	0.230	65	0.207	21
**↑C18:1-OH**	0.750	1	0.675	1

^1^ Ala, alanine; Arg, arginine; Cit, citrulline; Glu, glutamate; Gly, glycine; Leu, leucine, isoleucine and alloisoleucine, Met, methionine; Orn, ornithine; Phe, phenylalanine; Tyr, tyrosine; Val, valine; C0, free carnitine; C3, propionylcarnitine; C4, butyryl- and isobutyrylcarnitine; C5, isovaleryl- and 2-methylbutyrylcarnitine; C5:1, tiglylcarnitine; C3DC, malonylcarnitine; C4DC, methylmalonylcarnitine; C5DC, glutarylcarnitine; C4-OH, 3-hydroxybutyrylcarnitine; C5-OH, 3-hydroxyisovalerylcarnitine; C6, hexanoylcarnitine; C6DC, methylglutarylcarnitine; C8, octanoylcarnitine; C10, decanoylcarnitine; C10:1, decenoylcarnitine; C10:2, decadienoylcarnitine; C14, myristoylcarnitine; C14:1, tetradecenoylcarnitine; C16, palmitoylcarnitine; C16:1, palmitoleylcarnitine; C16-OH, 3-hydroxypalmitoylcarnitine; C18:1, oleoylcarnitine; C18:1-OH, 3-hydroxyoleoylcarnitine.

**Table 3:** Mean Ht values for the neonatal population of different gestational and post-natal ages. The SD was equal to 6 for all timepoints, approximately equal to one-fourth of the range of the data. Since most neonatal sampling takes place within the first 72 h of life, the mean Ht for days 0–3 was specifically considered, with day 0 being the date of birth.

Gestational Age	Post-Natal Age at Blood Collection (Days)							
	0	1	2	3	7	14	28	
**Term (35–42 weeks)**	53	51	50	49	45	42	38	**Mean Ht**
**Preterm (29–34 weeks)**	50	49	47	45	44	38	31	

## Data Availability

Data presented in this study are available on request from the corresponding author.

## References

[1] Wilken B., Wiley V. (2008). Newborn screening. Pathology.

[2] Jansen M.E., Metternick-Jones S.C., Lister K.J. (2016). International differences in the evaluation of conditions for newborn bloodspot screening: A review of scientific literature and policy documents. Eur. J. Hum. Genet..

[3] IJzebrink A., van Dijk T., Franková V., Loeber G., Kožich V., Henneman L., Jansen M. (2021). Informing Parents about Newborn Screening: A European Comparison Study. Int. J. Neonatal Screen..

[4] Sharma A., Jaiswal S., Shukla M., Lal J. (2014). Dried blood spots: Concepts, present status, and future perspectives in bioanalysis. Drug Test. Anal..

[5] Guthrie R., Susi A. (1963). A simple phenylalanine method for detecting phenylketonuria in large populations of newborn infants. Pediatrics.

[6] Mitchell M.L., Larsen P.R., Levy H.L., Bennett A.J., Madoff M.A. (1978). Screening for congenital hypothyroidism. Results in the newborn population of New England. JAMA.

[7] Chace D.H., Kalas T.A., Naylor E.W. (2003). Use of Tandem Mass Spectrometry for Multianalyte Screening of Dried Blood Specimens from Newborns. Clin. Chem..

[8] Schulze A., Lindner M., Kohlmüller D., Olgemöller K., Mayatepek E., Hoffmann G.F. (2003). Expanded newborn screening for inborn errors of metabolism by electrospray ionization-tandem mass spectrometry: Results, outcome, and implications. Pediatrics.

[9] Clague A., Thomas A. (2002). Neonatal biochemical screening for disease. Clin. Chim. Acta.

[10] Loukas Y.L., Soumelas G.S., Dotsikas Y., Georgiou V., Molou E., Thodi G., Boutsini M., Biti S., Papadopoulos K.J. (2010). Expanded newborn screening in Greece: 30 months of experience. Inherit. Metab. Dis..

[11] la Marca G. (2014). Mass spectrometry in clinical chemistry: The case of newborn screening. J. Pharm. Biomed. Anal..

[12] la Marca G., Carling R.S., Moat S.J., Yahyaoui R., Ranieri E., Bonham J.R., Schielen P.C.J.I. (2023). Current State and Innovations in Newborn Screening: Continuing to Do Good and Avoid Harm. Int. J. Neonatal Screen..

[13] Mian P., Flint R.B., Tibboel D., van den Anker J.N., Allegaert K., Koch B.C.P. (2017). Therapeutic Drug Monitoring in Neonates: What Makes them Unique?. Curr. Pharm. Des..

[14] Patel P., Mulla H., Tanna S., Pandya H. (2010). Facilitating pharmacokinetic studies in children: A new use of dried blood spots. Arch. Dis. Child..

[15] Autmizguine J., Benjamin D.K., Smith P.B., Sampson M., Ovetchkine P., Cohen-Wolkowiez M., Watt K.M. (2014). Pharmacokinetic Studies in Infants Using Minimal-risk Study Designs. Curr. Clin. Pharmacol..

[16] Howie S.R.C. (2011). Blood sample volumes in child health research: Review of safe limits. Bull. World Health Organ..

[17] European Medicines Agency (2009). Guideline on the Investigation of Medicinal Products in the Term and Preterm Neonate (EMEA/536810/2008). https://www.ema.europa.eu/en/investigation-medicinal-products-term-preterm-neonate-scientific-guideline#current-version-section.

[18] (2022). ICH Guideline M10 on Bioanalytical Method Validation and Study Sample Analysis-Step 5 (EMA/CHMP/ICH/172948/2019). https://www.ema.europa.eu/en/ich-m10-bioanalytical-method-validation-scientific-guideline.

[19] Denniff P., Spooner N. (2010). The effect of hematocrit on assay bias when using DBS samples for the quantitative bioanalysis of drugs. Bioanalysis.

[20] De Kesel P.M., Sadones N., Capiau S., Lambert W.E., Stove C.P. (2013). Hemato-critical issues in quantitative analysis of dried blood spots: Challenges and solutions. Bioanalysis.

[21] Capiau S., Veenhof H., Koster R.A., Bergqvist Y., Boettcher M., Halmingh O., Keevil B.G., Koch B.D.P., Linden R., Pistos C. (2019). Official International Association for Therapeutic Drug Monitoring and Clinical Toxicology Guideline: Development and validation of Dried Blood Spot-based methods for Therapeutic Drug Monitoring. Ther. Drug Monit..

[22] Jager N.G.L., Rosing H., Schellens J.H., Beijnen J.H. (2014). Procedures and practices for the validation of bioanalytical methods using dried blood spots: A review. Bioanalysis.

[23] Dvořák M., Ryšavá L., Kubá P. (2020). Capillary Electrophoresis with Capacitively Coupled Contactless Conductivity Detection for Quantitative Analysis of Dried Blood Spots with Unknown Blood Volume. Anal. Chem..

[24] Yu M., Dolios G., Yong-Gonzalez V., Björkqvist O., Colicino E., Halfvarson J., Petrick L. (2020). Untargeted metabolomics profiling and hemoglobin normalization for archived newborn dried blood spots from a refrigerated biorepository. J. Pharm. Biomed. Anal..

[25] Baillargeon K.R., Brooks J.C., Miljanic P.R., Mace C.R. (2022). Patterned Dried Blood Spot Cards for the Improved Sampling of Whole Blood. ACS Meas. Sci. Au.

[26] Delahaye L., Heughebaert L., Lühr C., Lambrecht S., Stove C.P. (2021). Near-infrared-based hematocrit prediction of dried blood spots: An in-depth evaluation. Clin. Chim. Acta.

[27] Daousani C., Karalis V., Malenović A., Dotsikas Y. (2019). Hematocrit effect on dried blood spots in adults: A computational study and theoretical considerations. Scand. J. Clin. Lab. Invest..

[28] Jopling J., Henry E., Wiedmeier S.E., Christensen R.D. (2009). Reference Ranges for Hematocrit and Blood Hemoglobin Concentration During the Neonatal Period: Data From a Multihospital Health Care System. Pediatrics.

[29] Lawson A.J., Bernstone L., Hall S.K. (2016). Newborn screening blood spot analysis in the UK: Influence of spot size, punch location and haematocrit. J. Med. Screen..

[30] Peng G., Tang Y., Cowan T.M., Zhao H., Scharfe C. (2021). Timing of Newborn Blood Collection Alters Metabolic Disease Screening Performance. Front. Pediatr..

[31] McHugh D., Cameron C.A., Abdenur J.E., Abdulrahman M., Adair O., Al Nuaimi S.A., Åhlman H., Allen J.J., Antonozzi I., Archer S. (2011). Clinical validation of cutoff target ranges in newborn screening of metabolic disorders by tandem mass spectrometry: A worldwide collaborative project. Genet. Med..

[32] Schmidt J.L., Castellanos-Brown K., Childress S., Bonhomme N., Oktay J.S., Terry S.F., Kyler P., Davidoff A., Greene C. (2012). The impact of false-positive newborn screening results on families: A qualitative study. Genet. Med..

[33] Mei J.V., Alexander J.R., Adam B.W., Hannon W.H. (2001). Use of Filter Paper for the Collection and Analysis of Human Whole Blood Specimens. J. Nutr..

[34] Holub M., Tuschl K., Ratschmann R., Strnadová K.A., Mühl A., Heinze G., Sperl W., Bodamer O.A. (2006). Influence of hematocrit and localisation of punch in dried blood spots on levels of amino acids and acylcarnitines measured by tandem mass spectrometry. Clin. Chim. Acta.

[35] Peng M., Liu L., Peng L. (2012). Evaluation of factors influencing accuracy in the analysis of succinylacetone in dried blood spots. Clin. Chim. Acta.

[36] Hall E.M., Flores S.R., De Jesús V.R. (2015). Influence of Hematocrit and Total-Spot Volume on Performance Characteristics of Dried Blood Spots for Newborn Screening. Int. J. Neonatal Screen..

[37] Butler A.M., Charoensiriwatana W., Krasao P., Pankanjanato R., Thong-Ngao P., Polson R.C., Snow G., Ehrenkranz J. (2017). Newborn Thyroid Screening: Influence of Pre-Analytic Variables on Dried Blood Spot TSH Measurement. Thyroid.

[38] Samardzic J., Turner M.A., Bax R., Allegaert K. (2015). Neonatal medicines research: Challenges and opportunities. Expert Opin. Drug Metab. Toxicol..

[39] Patel P., Tanna S., Mulla H., Kairamkonda V., Pandya H., Lawson G. (2010). Dexamethasone quantification in dried blood spot samples using LC-MS: The potential for application to neonatal pharmacokinetic studies. J. Chromatogr. B Analyt. Technol. Biomed. Life Sci..

[40] Patel P., Mulla H., Kairamkonda V., Spooner N., Gade S., Della Pasqua O., Field D.J., Pandya H.C. (2013). Dried blood spots and sparse sampling: A practical approach to estimating pharmacokinetic parameters of caffeine in preterm infants. Br. J. Clin. Pharmacol..

[41] Suyagh M., Collier P.S., Millership J.S., Iheagwaram G., Millar M., Halliday H.L., McElnay J.C. (2011). Metronidazole population pharmacokinetics in preterm neonates using dried blood-spot sampling. Pediatrics.

[42] De Vis J.B., Hendrikse J., Groenendaal F., de Vries L.S., Kersbergen K.J., Benders M.J.N.L., Petersen E.T. (2014). Impact of neonate haematocrit variability on the longitudinal relaxation time of blood: Implications for arterial spin labelling MRI. Neuroimage Clin..

[43] Widness J.A. (2008). Pathophysiology of Anemia During the Neonatal Period, Including Anemia of Prematurity. Neoreviews.

[44] Colombatti R., Sainati L., Trevisanuto D. (2016). Anemia and transfusion in the neonate. Semin. Fetal Neonatal Med..

[45] Saito-Benz M., Flanagan P., Berry M.J. (2020). Management of anaemia in pre-term infants. Br. J. Haematol..

[46] Widness J.A. (2000). Pathophysiology, Diagnosis, and Prevention of Neonatal Anemia. Neoreviews.

[47] Salsbury D.C. (2001). Anemia of prematurity. Neonatal Netw..

[48] Aher S., Malwatkar K., Kadam S. (2008). Neonatal anemia. Semin. Fetal Neonatal Med..

[49] Ramamurthy R.S., Berlanga M. (1987). Postnatal alteration in hematocrit and viscosity in normal and polycythemic infants. J. Pediatr..

[50] Pappas A., Delaney-Black V. (2004). Differential diagnosis and management of polycythemia. Pediatr. Clin. N. Am..

[51] Alsafadi T.R.M., Hashmi S.M., Youssef H.A., Suliman A.K., Abbas H.M., Albaloushi M.H. (2014). Polycythemia in Neonatal Intensive Care Unit, Risk Factors, Symptoms, Pattern, and Management Controversy. J. Clin. Neonatol..

[52] van Vliet K., van Ginkel W.G., van Dam E., de Blaauw P., Koehorst M., Kingma H.A., van Spronsen F.J., Heiner-Fokkema M.R. (2020). Dried blood spot versus venous blood sampling for phenylalanine and tyrosine. Orphanet J. Rare Dis..

[53] Edelbroek P.M., van der Heijden J., Stolk L.M. (2009). Dried Blood Spot Methods in Therapeutic Drug Monitoring: Methods, Assays, and Pitfalls. Ther. Drug. Monit..

[54] Haga M., Isobe M., Kawabata K., Shimizu M., Mochizuki H. (2022). The Acylcarnitine Profile in Dried Blood Spots is Affected by Hematocrit: A Study of Newborn Screening Samples in Very-Low-Birth-Weight Infants. Am. J. Perinatol..

[55] Moat S.J., George R.S., Carling R.S. (2020). Use of Dried Blood Spot Specimens to Monitor Patients with Inherited Metabolic Disorders. Int. J. Neonatal Screen..

[56] Foivas A., Malenović A., Kostić N., Božić M., Knežević M., Loukas Y.L., Dotsikas Y. (2016). Quantitation of brinzolamide in dried blood spots by a novel LC-QTOF-MS/MS method. J. Pharm. Biomed. Anal..

[57] R Core Team (2020). R: A Language and Environment for Statistical Computing.

[58] Esan A.J. (2016). Hematological differences in newborn and aging: A review study. Hematol. Transfus. Int. J..

[59] Rohatgi A. (2022). Web Plot Digitizer. https://automeris.io/WebPlotDigitizer.

[60] OnlineStatBook Version 2.0. Normal Distribution Calculator. http://onlinestatbook.com/2/calculators/normal_dist.html.

[61] Manta-Vogli P.D., Schulpis K.H., Loukas Y.L., Dotsikas Y. (2020). Perinatal free carnitine and short chain acylcarnitine blood concentrations in 12,000 full-term breastfed newborns in relation to their birth weight. Pediatr. Neonatol..

